# Secondary traumatic stress in iranian midwives: stimuli factors, outcomes and risk management

**DOI:** 10.1186/s12888-022-03707-7

**Published:** 2022-01-24

**Authors:** Maryam Hajiesmaello, Sepideh Hajian, Hedyeh Riazi, Hamid Alavi Majd, Roya Yavarian

**Affiliations:** 1 Student Research Committee, Department of Midwifery and Reproductive Health, School of Nursing and Midwifery, Shahid Beheshti University of Medical Sciences, Tehran, Iran; 2 Department of Midwifery and Reproductive Health, School of Nursing and Midwifery, Shahid Beheshti University of Medical Sciences, Tehran, Iran; 3 Department of Midwifery & Reproductive Health, School of Nursing & Midwifery, Shahid Beheshti University of Medical Sceinces, Tehran, Iran; 4 Department of Biostatistics, School of Allied Medical Sciences, Shahid Beheshti University of Medical Sciences, Tehran, Iran; 5 Department of Psychiatry, Urmia University of Medical Sciences, Urmia, Iran

**Keywords:** Secondary traumatic stress, Midwife, Job experience

## Abstract

**Background:**

The present qualitative study was conducted to explain the experiences of secondary traumatic stress (STS) and its related factors in midwives working in maternity wards.

**Methods:**

Data were collected using semi-structured interviews with 11 midwives working in the maternity wards of hospitals in Urmia, Iran, through in-depth interviews with open-ended questions. Data were analyzed using the conventional content analysis approach.

**Results:**

The results of data analysis led to the extraction of three themes, seven main categories, and 18 subcategories. The first theme was “STS stimuli,” with the two categories of “Discriminatory approach to midwifery” and “The nature of the midwifery profession”. The second theme was “Traumatic outcomes”, which included the subcategories of “Psychological-emotional trauma”, “Physical trauma” and “Social trauma”. The third theme was “Risk management”, which had the two subcategories of “Reactive approach” and “Proactive approach”.

**Conclusions:**

The results showed that, in addition to the traumatic nature of events that midwives experience during work as the secondhand victims, factors such as governance-organizational structure, unbalanced distribution of power, and poor supportive laws undermine their professional role and provide conditions conducive to STS. Therefore, avoiding traumatic situations and scientific and skill self-empowerment were the most important strategies adopted by the midwives in this study to prevent risky situations and cope with the consequences of STS. The participation of midwifery stakeholders in policy-making and adopting supportive legislation in redefining the position and role of midwives can play a major role in reducing STS and sustaining their role and position in maternal care.

## Introduction

Midwifery has a history as old as humanity, and a midwife is a professional and responsible person who, with the cooperation of women, provides the needed care and support during pregnancy, childbirth and the postpartum period and also the care needed for the newborn and child[1]. Since midwives are at the forefront of healthcare delivery in the healthcare system, they face numerous occupational problems and hazards every day that lay the ground for physical and psychological stress. Challenging incidents and irreversible complications, such as the death of the mother or child during prenatal care, labor, and delivery can make midwives susceptible to burnout, fatigue, and psychological-emotional disorders such as secondary traumatic stress (STS).

STS is an occupational threat to healthcare providers caring for injured patients [2]. According to Figley, STS is a natural consequence of the stress experienced when helping or wanting to help a suffering or injured patient [3].

Occupational accidents in midwifery include not only emergencies, but also violent behavior by other healthcare providers, including physicians, and any disrespectful behavior by pregnant women and their families [2, 4]. Damages following childbirth trauma have growing and lasting effects not only on the parturient, but also on those present at the scene of the traumatic event, including the spouse and healthcare providers [5]. Furthermore, a hallmark of the midwifery profession is that it entails high levels of empathy with the woman in labor, as midwives establish a very close contact with these women; consequently, midwives have the power to experience what the mother is experiencing, and this distinguished attribute thus exposes midwives to STS [6].

### Conceptual framework

The two important factors of exposure to and awareness of frequent traumatic events can expose people to STS and sometimes lead them to quitting their job [2]. The concept of STS should be distinguished from the concept of Post-Traumatic Stress Disorder (PTSD). In PTSD, after having been in a very traumatic situation, certain symptoms manifest themselves that sometimes take the form of direct experience of the traumatic event, such as death or its threat or serious traumas, and sometimes take the form of an indirect experience, such as witnessing an event or hearing about what has happened to someone else [7]. The characteristic symptoms of PTSD are classified into four main groups, including intrusion, avoidance, arousal, and negative alterations in cognition and mood.

Intrusion symptoms refer to the recurrent reminding of the traumatic event and distressing dreams. Avoidance symptoms refer to the deliberate avoidance of any situation, thoughts, or feelings that are somehow related to the traumatic event. Increased arousal symptoms include the lack of sleep, excessive excitement, irritability or anger, and difficulty concentrating. Negative alterations in cognition and mood include difficulty remembering the details of the accident, persistent negative emotions such as guilt and panic, persistent inability to experience positive emotions, or decreased interest in important life activities, and distorted beliefs about the cause or effects of the accident [7].

STS has symptoms similar to PTSD but is created by contact with people who have been directly exposed to trauma and stress [8]. It can be argued that the only difference between these two disorders is that, in STS, the subject does not directly experience being in a traumatic situation; rather, another traumatized person is perceived as a traumatic situation for the second person, and that second person becomes an indirect victim of the trauma. Healthcare providers may not directly experience a traumatic event that actually occurs for the patient, but they can be a “secondhand” traumatized person who has witnessed the trauma, and the injured person is then the “firsthand” traumatized person. Therefore, STS is also defined as natural behaviors and emotions following learning of a traumatic event experienced by others [9].

Learning of the traumatic events that have happened to another person can also cause trauma. When a stressful event occurs for one person, this stress can be disseminated in the organization, affect other individuals in the organization or group and create STS in them [3]. STS usually occurs suddenly without warning signs and in connection with the two concepts of empathy and exposure, and is therefore sometimes referred to as “compassion fatigue” in literature [9].

Several studies have reported STS symptoms in social workers [10], hospice nurses [11], nurses working in departments such as hospital emergency rooms [12], oncology departments [13], intensive care units [14] and pediatric wards [15]. Despite the prominence of STS in numerous studies and professional groups of healthcare providers, the existence of unique aspects in midwifery limits the generalizability of the results of these studies to midwives.

Leinweber & Rowe reported the emergence of STS as the first warning sign about the effects of continuing to care for women in labor on midwives [6]. This exposure can have negative effects on midwives’ mental health, undermine their quality of work life and family life, and lead to PTSD in some cases [16].

According to research, 26.9-42% of midwives and midwifery nurses working in labor and delivery units reported moderate to severe symptoms of STS [2, 17]. Teffo also reported moderate degrees of STS in midwives [18]. Some qualitative studies also showed that during traumatic deliveries, midwives feel guilty, ashamed, and blamed for the burden of responsibility resulting from a high-risk delivery process and suffer from a sense of inability to protect the mother. These feelings make them very vulnerable to STS [4, 5, 19].

The manifestation of symptoms of PTSD in midwives is a significant issue, as it can have potentially negative consequences. These symptoms gradually decrease in some midwives, but in others, they reduce the ability to adapt in the future, or lead to job burnout and a cycle of more errors [20] or impaired empathy and emotionless care [21]. Therefore, in some cases, midwives do not approach the delivery process as a physiological phenomenon and inevitably make unnecessary interventions to avoid the occurrence of traumatic events [4, 5, 22].

Iran is one of the developing countries that have made noticeable progress in reducing maternal mortality [23]. Most deliveries are performed in hospitals by midwives under the supervision of obstetricians. Generally, no nurses are present in labor and delivery wards, and midwives provide all maternal cares. In each work shift, one midwife, who is in charge of the shift, supervises other midwives.

Despite the over-century-long history of midwifery as a profession, the medicalization of midwifery care over the last three decades is common due to the physician-centered approach to labor and delivery care, particularly in private and teaching hospital. As such, in one study, only 35% of deliveries were performed by midwives, 65% by obstetricians, and 93% of the mothers had been hospitalized before labor pains began[24]. A report from public hospitals in Iran shows an increasing trend in cesarean section rates from 1979 to 2009 [25]. In recent years, 48% of deliveries are cesarean section. In some none public hospitals, this rate reaches 87% of deliveries. This leads to the midwife moving away from physiological care and independent performance of low-risk deliveries; the result of this transformation has been the dissatisfaction of many midwives [25]. Nonetheless, in some rural areas that do not have quick access to hospitals, there are delivery centers that provide midwife-centered services and where low-risk deliveries are performed by midwives. The results of two studies in Iran showed that nearly two-thirds of midwives under study in Sanandaj reported depressive symptoms and 65% reported moderate to severe emotional fatigue [26]; and more than two-thirds of the midwives working in hospitals in Mashhad also reported experiencing moderate to severe stress due to workplace accidents [27].

Yet, no studies in Iran have addressed the experiences of midwives in dealing with occupational problems from this perspective, and many aspects related to mental stress and the causes of its occurrence or exacerbation in midwives working in maternity wards remain unknown and neglected. The present study explained midwives’ experiences of STS and its various dimensions in some midwives working in labor and delivery units of specialty hospitals in Urmia, the capital of West Azarbaijan Province in Iran, to discover the consequences, related factors, and means of coping with this issue among them in depth.

## Methods

### Study design

The present study was a qualitative research with a content analysis approach that was conducted in 2020 over a period of nine months.

### Samples and recruitment

The participants of this study were midwives working at hospitals who met the following inclusion criteria: Having a bachelor’s or higher degree in midwifery, work experience in the labor and delivery department, experience in dealing with labor traumas while delivering midwifery services, and the ability to understand and express their experiences and transfer them to the researcher. The exclusion criterion was the participant’s refusal to continue participating in the study at any time.

The participants were selected via purposive sampling with maximum variation of demographic characteristics and a high potential for providing rich information. They were selected from the maternity wards in teaching, non-teaching, and private hospitals and a birth center with midwife-centered services in Urmia. All the interviews were conducted by the first author. She is a faculty member and Ph.D. student majoring in reproductive health .

### Data collection

After obtaining the code of ethics and sampling permits, data were collected through in-depth interviews with open-ended questions with the eligible participants. After selecting the participants, necessary explanations about the study objectives were provided to them by phone or in person and their willingness to participate in the research was ensured, and after obtaining their written consent, arrangements were made about the time and place of the interviews with the agreement of the researcher and participants.

All the interviews were recorded with the permission of the interviewees and the participants were reassured about the anonymity and confidentiality of their information and the lack of access of anyone other than the researcher to the audio files. The interviews began by filling in a personal and occupational information checklist with items related to the research objective and by asking an open-ended question:

“Please describe your experience of an adverse event, such as a traumatic delivery or any traumatic event related to labor care that you have witnessed”. Then, the participants were asked for further explanation and details about some ambiguous parts of their statements through probing questions. Table 1 presents the interview guide. During the interviews, non-verbal information, such as facial expressions and feelings of the interviewees (anger, sadness, emphasis, feeling tearful, etc.) were recorded by field notes. The interviews lasted 45-75 min. At the end of each interview, after listening to the audio files, the interviews were promptly transcribed and the text was analyzed before the next interviews.

**Table 1:** Semi-structured interview guide

Please describe your experience of an adverse event, such as traumatic delivery or any traumatic event related to labor care that you have witnessed.
How did you feel after that event?
What effects did the event have on you or your relationship with the parturient, her family, or your colleagues?
When did these complications start and how long did they last?
How did you seek to cope with this problem and its reminders?
Did the event change the nature of your next professional decisions?
What factors do you think influenced you in developing these symptoms?

The interviews continued until data saturation, which occurred with the ninth interview. Two additional interviews were conducted to ensure data saturation, and the data from 11 interviews were reviewed. The sampling process lasted from April to December 2020.

### Data analysis

Data were analyzed using the conventional content analysis approach based on the steps proposed by Graneheim & Lundman [28]. After listening to each audio file twice, the whole text of the interview was transcribed verbatim in Microsoft Word. After reading the interview text several times to get a general understanding of its content, first, an initial coding of all the interview texts was carried out as the units of analysis. Then, MAXQDA v.10 software was used for the classification of the similar codes and transforming them to the final codes, their integration into the subcategories and main categories, and finally the extraction of the themes.

### Rigor and trustworthiness

This study used the four criteria of Guba & Lincoln [29]. The following measures were taken to ensure the credibility of the data: Long-term engagement with the data, member check, independent code checking and data classification by the research team and then reaching agreement on the codes and categories and themes. The code-recode method was used to ensure dependability. Two weeks after the initial coding of two of the interviews, the researcher again coded the two interviews and revised a few of the codes. An experienced researcher outside the research team was also asked to review the codes and classifications and offer feedback. The transferability of the data was ensured by sampling with maximum variation of personal and demographic characteristics and place of work. Efforts were made to explain the sampling and interview stages and participants’ characteristics in a way that other researchers would also have a correct understanding of the work process if they needed to conduct similar studies. Also, the opinion of two researchers experienced in qualitative studies on midwifery were sought to evaluate the results, and some textual data were used to achieve the confirmability of the results.

## Results

 The mean age of the participating midwives was 37.09 (8.50) years, and their mean years of employment in maternity wards was 12.54 (8.11). Among the participants, seven had a bachelor’s degree and three a master’s degree in midwifery and one was a PhD student in this discipline. Most participants were married (63.6%) and over half of them had one or two children (55.5%). Table 2 shows the demographic and personal information of the participants. All the participants allowed the interviews to be audio recorded. Each participant was interviewed in one session, except for one interview, which remained incomplete as the work shift was over and required another session. Based on a previous appointment, the researcher contacted this participant again to determine the time and place of the second interview session with her, but she refused to continue, and upon her request not to disclose her statements at all, the transcript of the first session of the interview with her was also deleted and excluded from the study.

**Table 2:** The personal and demographic information of the midwives participating in the study

Participant number	Age	Work experience (year)	Place of work	Midwifery degree	Marital status	Number of children	Type of trauma occurred
P1	46	22	Public teaching hospital	Master’s degree (Midwifery Instructor)	Married	2	Rupture of the uterus and fetal death- Disrespectful behavior
P2	28	8	Public teaching hospital	Master’s degree (Midwifery Instructor)	Single	-	Low grade Apgar and neonatal death
P3	46	21	Non-teaching public hospital	Bachelor’s degree	Married	2	Shoulder dystocia- perineal rupture(Grade 3)
P4	31	8	Non-teaching public hospital	Bachelor’s degree	Married	1	Stillbirth- shoulder dystocia
P5	34	5	Public teaching hospital	Master’s degree (Midwifery Instructor)	Single	-	Disrespectful behavior- Rupture of the cervix
P6	38	14	Birth center(rural)	Bachelor’s degree	Married	-	Shoulder dystocia – internal bleeding in mother
P7	24	1	Non-teaching public hospital	Bachelor’s degree	Single	-	Postpartum hemorrhage and hysterectomy
P8	30	3	Non-teaching public hospital	Bachelor’s degree	Married	2	Clavicle fracture
P9	41	16	Public teaching hospital	PhD student (Midwifery Instructor)	Single	-	Shoulder dystocia- Disrespectful behavior
P10	51	25	Non-teaching public hospital	Bachelor’s degree	Married	2	Witness numerous events in labor and delivery
P11	39	15	Non-public hospital	Bachelor’s degree	Married	1	Postpartum hemorrhage

**Fig. 1:**
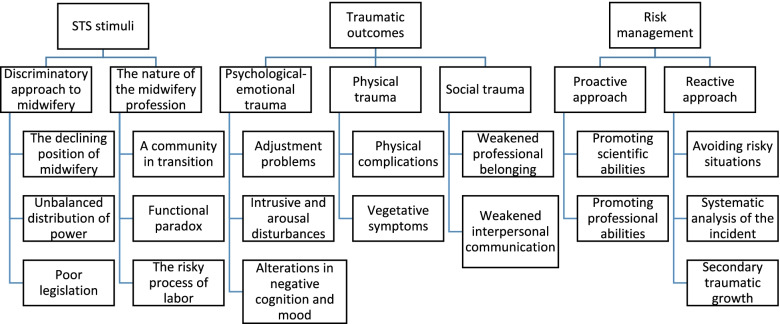
Conceptual model of the themes, categories and subcategories of secondary traumatic stress in Iranian midwives

After removing the duplicate codes, the 65 final codes extracted from the interview texts were classified into 18 subcategories, seven categories, and three themes (Fig. 1). “STS stimuli”, “Traumatic outcomes” and “Risk management” were the main themes of this study.

### STS stimuli

This theme had two main categories, including discriminatory approach to midwifery and the nature of the midwifery profession, each with three subcategories. This theme consists of categories that make midwives prone to STS.

### Discriminatory approach to midwifery

Midwives often believed that the discriminatory approach of the healthcare system to midwifery puts them at risk of STS in the face of adverse events in the workplace. This category had the following three subcategories.

### The declining position of midwifery

Most midwives believed that their job position in the maternity ward was unclear. They did not have autonomy in making decisions about the mothers to whom they provided care. Their professional experiences were neglected, and they complained about a lack of job control.

“We have no autonomy. We are neither a nurse, who is comfortable and just follows the physician’s instructions, nor are we completely independent. There are no longer any good midwifery days now” (P10).

“The professional circumstances of midwifery have deteriorated. Midwives do not dare make any comments on the patients’ state. You think you have no power” (P1).

### Unbalanced distribution of power

The obligation to follow the more experienced colleagues and being under pressure in traumatic situations were extracted from the young interviewees’ statements. Also, the unbalanced distribution of power between obstetricians and the midwives engaging in labor care in the maternity ward laid the ground for shifting responsibility and negligence towards them [the midwives]. An interviewee said:

“I told the shift manager that the fetus is large and the mother can’t have a vaginal delivery and I won’t do it. She said ‘You have this responsibility and you should learn to not disagree with the shift manager’. Then when the baby came out and his hand dropped like this, I began crying before the mother did. They did not trust me because of my little work experience. The physician also trusts her. They paid no heed to my words” (P4).

Some midwives also stated that they had to get the physician’s consent to gain some benefits that they considered rightfully theirs.

“Previously, there was some collusion between the midwives and the obstetricians, either out of fear, or because of a weakness the midwife might have had; so, to avoid the problem being reported to the authorities, she had to cooperate with the physician like that” (P10).

### Poor legislation

Most participants believed that ignorance about their legal rights would lead to fear in the case of adverse events. Some also complained of the non-accountability of the superiors, whether the midwife in charge of the shift or the resident, and stated that even though they were responsible for deciding about the delivery, in the case of accidents, responsibility fell on the midwife. Besides, midwives do not have proper union support and the current legislation poorly supports midwifery professionals and the stakeholders of this field are not involved in legislative processes. Several interviewees commented:

“When there’s a complication in childbirth, we are the first defendants on the line. There is no barrier or protector to help us not engage directly with the clients. We must attend the courts ourselves. Hospitals do not insure us. We must always take responsibility on our own” (P10).

“In very serious cases, such as the case involving maternal death, the physician didn’t support my colleague and claimed that ‘she had not informed me [the physician] from the beginning’, which ended up having bad consequences for my colleague” (P8).

“If it were up to me, it might not have happened. The shift manager applies fundal pressure and does not write anywhere that I (the delivery agent) am involved” (P4).

### The nature of midwifery

Midwifery is a profession with high responsibility and accountability, and the unique aspects of this profession, such as empathy and having a care approach emphasizing physiological delivery, on the one hand, and the unpredictability of some midwifery accidents, emergency medical interventions, and exposure to the clients’ expectations, on the other hand, are considered factors involved in creating STS in midwives. This category had three subcategories, as follows.

### Functional paradox

Despite the emphasis on evidence-based care approaches, which often recommend the physiological approach and the provision of services based on the individual needs of the mother, the decision-making conditions are occasionally not as they are expected to be. In this regard, some participants stated:

“The problem with our work is that our work has become a combination of physiological delivery with the new fully medicalized method” (P6).

“Childbirth is a difficult job; it becomes even more difficult if there is a complication. Being mistreated by the mother’s family even slightly; this is a profession that deals directly with clients from all classes with any and all cultural backgrounds” (P10).

Furthermore, many midwives believed that there was a conflict of interest between midwives, specialist physicians, and obstetrician-gynecologist residents that affected midwives’ paradoxical function.

“[Physicians and residents] know that if midwives work well and scientifically, the number of natural deliveries will increase and cesarean sections will decrease and their market will shrink; so, they oppose us” (P9).

### A community in transition

Over time, changes have developed in the attitude toward the importance of childbearing. Most families have fewer members and will bear one or at most two children. One of the participants discussed this matter and said:

“Families have a smaller size these days. They’ll bear one or at most two children. Families’ expectations have risen, and rightfully so. They want everything to be done without compromising the mother’s privacy” (P3).

Moreover, the expectations of midwifery service recipients and their media literacy have also changed. When women hear a scientific term from a midwife, they immediately begin to collect information about it on the web. Also, their knowledge of the laws has grown; therefore, they seek legal action with the slightest mistake. One participant described the changing needs and expectations of women in this area as follows:

“Mothers are reluctant to give birth in teaching hospitals, except for ones with high-risk pregnancies or those who have been referred there, while in the past, this was not the case. For example, the mother sees that someone who gives birth in a private hospital can enjoy the company of her mother or husband at birth, while the presence of the mother’s relatives is prohibited in teaching hospitals, and the mother is deprived even from having her cellphone on her. The mother can’t communicate with her family. This gap between the expectations and the infrastructure bothers both the mother and the midwifery personnel” (P9).

### The risky process of labor

Midwives believed that the process of pregnancy and childbirth, despite being physiological, could be potentially dangerous and hazardous to both mothers and midwives. Some of these risks are related to underlying maternal causes that make a particular pregnancy and childbirth risky. The full awareness of the mother’s caregivers and delivery agents about the mother’s conditions can keep them alert and help prevent irreparable accidents through the adoption of appropriate measures. Nonetheless, some accidents and complications during childbirth are unpredictable and midwives should always be prepared to deal with such incidents.

“The mother gave birth, and I controlled her bleeding until 2 hours later. She did not have bleeding but had a constant drop in blood pressure. An ultrasound was taken but showed nothing. They had to perform a laparotomy on the mother, and after diagnosing bleeding from the internal varicose veins, the mother eventually underwent a hysterectomy” (P6).

“Not all cases are always without problems. It happens that we have taken a history and provided care for some hours, but the mother has not mentioned that she has had a postpartum hemorrhage in her previous delivery” (P9).

### Traumatic outcomes

The most important aspect of harm to midwives following adverse events in the maternity ward is the occurrence of traumatic physical, psychological, and social outcomes, which were mentioned by all the participants in this study. Outcomes are classified into three categories and seven subcategories.

### Psychological-emotional traumas

All the participants stated that disturbing psychological-emotional traumas were a consequence of their witnessing traumatic deliveries that have disturbed them for a long time. These symptoms often manifested in them in the form of adjustment problems, Intrusive and arousal disturbances, negative alterations in cognitions and mood.

### Adjustment problems

Some of the interviewees discussed disappointment with the prospects of improvement in their professional conditions, their uninformed choice of the profession, and regret about having chosen this profession:

“Sometimes I felt extremely regretful about why my job is so risky! Why did I choose this field?” (P3).

“I started midwifery with love and passion. You can get disappointed with any job, but this (the lack of support following traumatic events) makes you hate your job. I always said I wished I hadn’t chosen this field” (P4).

### Intrusive and arousal disturbances

The participants said they experienced several intrusive and arousal episodes including mental stabilization of the trauma, obsessive-compulsive disorder, rumination, fear of trauma being repeated, sadness and crying, decreased self-esteem, hypervigilance and irritability.

“I cried day and night. It got on my nerves (P4).

“For five whole years, it was as if that event was always following me like my shadow” (P3).

“I kept saying to myself that it must have been my fault and I wished I had paid more attention to the patient, I wished I had not let anyone touch my patient. This issue had penetrated my brain and I couldn’t sleep” (P7).

“We have a colleague who was very afraid of CP. When the fetus stays behind the perineum and develops slight bradycardia, she gets very scared. She herself rushes and then she rushes everyone else too, and transfers the stress to everyone” (P8).

### Alterations in negative cognition and mood

Many participants stated that the incident had created intense fear in them, and that they had distorted thoughts about the cause of the accident afterwards, and felt guilty and remorseful, and constantly blamed themselves.

“After the incident, whenever I closed my eyes, I only heard that sound. Nothing had happened to the child, but it had caused great fear in me” (P8).

“After the hysterectomy, when I came out of the operating room, I saw the young husband of the patient saying, ‘I don’t want her anymore, she is of no use as a wife anymore’. This affected me a lot. I told myself that it must have been my fault; I wished I had paid more attention to the patient; I wished I had not let anyone touch my patient’” (P7).

“The impact of what happened, although it was four or five years ago, has lingered on to this day, mostly as a feeling of guilt” (P9).

### Physical traumas

The physical effects of adverse events in addition to psychological effects cause health dysfunction in those experiencing STS. This category includes the two subcategories of physical complications and vegetative symptoms.

### Physical complications

Several midwives believed that the recurrence of such events caused symptoms such as fatigue and some physical complications.

“Sometimes when I come home, I like to turn off the lights and not hear too much noise in the house, because there has been a lot of noise at work” (P8).

“After this incident, I developed skin problems, hair loss, and itchy skin” (P6).

### Vegetative symptoms

Most midwives stated that they had anorexia and weight loss, insomnia, and nightmares after the incident.

“I was very upset until I was acquitted. I lost my appetite. I thought to myself that maybe I was to blame too” (P3).

“It [the event] made me lose 4 kilos” (P4).

“I couldn’t sleep. When I went to bed, different thoughts came over me; I’d lost my appetite” (P7).

“I kept dreaming about that newborn. He [the baby] had started crawling on all fours and kept turning back and looking at me and talking to me” (P8).

### Social traumas

In addition to the mentioned traumas, most midwives believed they had experienced social traumas after what had happened in the maternity ward. Weakened professional belonging and weakened interpersonal communication were the two subcategories of this category.

### Weakened professional belonging

One of the complications resulting from the incident was the blemished professional records of the participants, and they had to struggle to keep their position due to the regulations governing the maternity ward. Moreover, distrust and getting disappointed about receiving peer support contributed to the formation of such outcome.

“These conditions are now accepted and I have to keep up in this bad situation and move forward. After all, we are part of this system, they can’t remove us. But what kind of job is this? How much should we struggle?” (P5).

“Naturally it [the incident] affects you. At the very least you get disappointed about your colleagues and know you can’t count on them anymore. I think to myself that if an accident was to happen now, my colleague wouldn’t support me, and this impacts my work” (P4).

### Weakened interpersonal communication

Some interviewees believed that the professional issues occurred in the workplace not only weakened their relationship with their colleagues and friends, but also affected their family relations. They had to spend a lot of energy to avoid these issues impacting their quality of family life, which caused even greater fatigue.

“I had become very aggressive with my friends and roommates. I had gotten nervous” (P7).

“When you see a bad attitude in the workplace, it unconsciously affects your life and your treatment of your children and affects your relationships and quality of life. And you should play pretend for your family to not learn of how much work pressure you’ve endured” (P1).

### Risk management

In this study, the midwives’ main response to the traumatic events experienced was either passive/ reactive or active/proactive. With the heading of risk management, these responses are divided into two categories, the reactive approach, with three subcategories, and the proactive approach, with two subcategories.

### Reactive approach

The reactive approach is based on responding to events after they occur. This approach is passive and retrospective and is based on the analysis of what has happened in the past to avoid a repetition of similar incidents in the future. This approach includes the three subcategories of avoiding risky situations, systematic analysis of the accident, and secondary traumatic growth.

### Avoiding risky situations

Some midwives said that, after the incident, they tried to justify themselves in some way, learn from the incident and avoid situations where the incident could happen again.

“I was upset myself. Then I said, ‘Oh, I did my job right. Everyone in my place would have done the same’” (P4).

“Not only in this incident, but in each and every case that happens, the first thing that comes to mind for everyone is that ‘I don’t want to get into trouble’” (P6).

### Systematic analysis of the incident

Most midwives said that they had tried to review the details of the incident and investigate its causes after it had occurred. They had tried to review in their mind any unsafe act and pass on these experiences to their other colleagues. Also, by reviewing and analyzing the details of their other colleagues’ experiences, they prepared to deal with such incidents and to act more intelligently in the face of similar situations.

“I asked myself where the mistake had been. Had I made a mistake? Did the mother have a problem? Was the fetus large, leading to a fourth-degree tear?” (P9).

“Cases examined in the committee on the mortality and morbidity of mothers are available in the system, and I read all of them, but some colleagues do not have much patience and time to study these cases” (P7).

### Secondary traumatic growth

An important aspect after the incidence of an adverse event was the pleasant feeling the participants experienced in situations where the patient had been rescued, and they felt gratitude and appreciation for these alarming situations, especially in near-miss events. Several participants stated:

“I try not to remind myself much of these problems at the patient’s bed. I try to tell the patient to think of good things as well, since this reduces my stress and increases my self-confidence” (P8).

“It was a pleasant experience in that I was very happy we had been able to save the patient. Maybe this incident helped prevent larger events, because I usually was so reckless” (P6).

“Thank God I did and wrote [the incident and measures report] correctly. Everything was right and then I was rewarded” (P7).

### Proactive approach

This approach is an active preventive approach that includes planning for the future so that problems do not arise. After the experience of these incidents, the midwives tried to identify future threats and prevent them by taking the necessary measures and planning, so that they would not face bigger problems in the future, which, from their point of view, can be achieved by upgrading their scientific and professional abilities. This approach consists of the two subcategories of upgrading scientific abilities and upgrading professional abilities.

### Upgrading scientific abilities

To prevent adverse events and the traumas caused by them, most midwives believed they should update their midwifery knowledge and commit themselves to follow evidence-based policies and protocols. They also believed that sharing the successful experiences of their peers helped other colleagues benefit from their experiences, which would then be effective in preventing future accidents.

“I always emphasize that if our scientific base is good, our performance is up to date and we do our job properly, then no one can blame us” (P9).

“These [events] made me study my lessons harder. And also read more about my rights and entitlements” (P1).

### Upgrading professional abilities

Another preventative measure according to the midwives was to try to increase their professional abilities. Most midwives said that caution and continuous care played an important role in the prevention of adverse events. Some of them said that it is best to hold regular meetings within the department and discuss the incidents. Midwives should also try to record and document all their activities and actions toward the mother. Following the experience of incidents, midwives tried to increase their communication with the patients and express sympathy toward them. They believed that this would reduce their stress.

“We have an intra-departmental meeting each month, and we review issues and errors or danger signs without addressing anyone in particular, so that no errors occur again later. Also, in these meetings, they acknowledge any peers who have prevented adverse events by timely diagnoses and actions” (P10).

“I think the most important thing we learned was to keep a well-maintained record of everything that we do” (P11).

## Discussion

STS stimuli, their traumatic outcomes, and risk management were three important aspects of midwives’ experience of STS in the present study.

### STS stimuli

The declining position of midwives and their vague professional perspectives were of the main concerns of midwives and a factor that made them vulnerable to accidents in the maternity ward. In recent years, due to the atmosphere prevailing in delivery rooms in Iran, which involves the dominance of physicians, unbalanced distribution of power and weakness in legislation and the lack of union support, the role of midwives in guiding childbirth has diminished as they experience a lack of job control. Meanwhile, studies have shown that having job control enables decision-making and autonomy and provides the opportunity to perform tasks independently[30, 31]. In a study in Cyprus, midwives found themselves in a difficult position in terms of supporting mothers and childbirth due to the dominance of medicine in the healthcare system, the physician-centered approach to childbirth, and the lack of sufficient organizational support [32]. In such system of medical paternalism, due to the lack of professional independence and the limited opportunities to gain experience in midwifery care in high-risk situations, there is potential for the emergence of stressful instances, including STS [33].

Furthermore, the particular nature of midwifery makes for another stimulus for STS in midwives. Despite the fact that midwives can play an important role in supporting natural childbirth, in Iran and some other countries [32], the midwifery model of natural childbirth has not been fully recognized and implemented. Even in public hospitals, the role of midwives is not recognized, and the continued presence of obstetricians has reduced the sense of professional independence in midwives. In a situation where midwives work without professional independence and under the supervision of obstetricians even for basic practices, they lose their abilities, and medical interventions in natural childbirth grow in number [34], and natural childbirth also acquires a medicalized approach. In this approach, which is known as the medical care model, birth is only normal in hindsight, but the medical management of pregnancy, labor, and delivery is essential for achieving the desired positive results in this care model [35]. Meanwhile, the midwifery care model is based on principles that state: Pregnancy and childbirth are a natural and important stage in a woman’s life; midwives can provide ongoing care to mothers; midwifery is an autonomous profession; and midwives provide woman-centered care [36]. In Iran, on the one hand, efforts have been made to empower midwives by promoting natural childbirth and setting evidence-based policies; on the other hand, with the dominance of the medicalized approach, the opportunity for evidence-based practice is taken away from midwives. This duality has led to a functional paradox in midwives. Also, the views of the public have shifted in recent years from a traditional to a modern approach, with consequences including reduced fertility, limited birth rates and the seeking of pregnancy and childbirth care in modern platforms, which can lead to women’s doubt about the appropriateness and sufficiency of midwives’ knowledge, skills and abilities [37]. The results of a qualitative study on midwives suggested that they were caught between the two philosophies of midwifery care and medicine and believed that when childbirth was managed by physicians, nothing would be left for midwives to do [4]. The realm of childbirth is controlled by networks of economic, social, and legal power [38]. Such a conflict of interest and paradox cause harm to midwives. Part of the cause of the “turf wars” between physicians and midwives in the maternity ward is the power given to them by the medical care model, which has caused an imbalance of power between the two professions and resulted in feelings of fear and weakness [4, 39]. In other studies, midwives stated that the lack of respect for their knowledge, skills and experience in facilitating labor and delivery and the ignoring of their clinical decision-making power cause frustration, dissatisfaction and distress in them [4, 22, 40].

The risky process of labor and delivery was another subcategory of the nature of midwifery in this study that exposed midwives to STS. Sometimes, extremely stressful events occur during routine care in labor and delivery which not only affect the mother and baby physically and emotionally, but can also affect any present midwives in the long run. The narrow border between natural and non-natural childbirth, and sometimes between life and death, exacerbates the drama of the threatening situation for midwives. Unpredictable events require a quick assessment of the situation, immediate decisions, such as calling a physician and preparing for possible interventions. Proper evaluation and appropriate interventions are important aspects of a midwife’s professional behavior. Nonetheless, when a physician is called to manage a case, a sense of inconsistency between the midwives’ accountability and the physicians’ authority emerges that challenges the midwives’ sense of competence [41].

### Traumatic outcomes

In the present study, the occurrence of psychological, emotional, and physical responses as part of the theme of traumatic outcomes of adverse events in midwives affected not only their professional but also personal life and even caused social harms and weakened their interpersonal relationships. In recent years, occupational mental health researchers have focused mainly on the negative long-term effects of job characteristics, especially chronic workplace-related stressors, such as the consequences of overwork, role conflicts, and inadequate organizational support, because these stressors are associated with various consequences in the physical and mental dimensions of employees’ health, such as fatigue and absenteeism[42]. Symptoms such as guilt and remorse, despair, sadness, and changes in self-confidence following exposure to occupational midwifery incidents have been observed both in this study and in similar studies [16, 17, 43–45]. These acute emotional responses to dealing with events may affect the subject’s professional perspective in addition to her personal life for days or even years [46]. In trying to deal with such incidents, midwives often think about the boundaries of accountability, professionalism, and their position among their colleagues and other healthcare providers. Under such circumstances, they may suppress their emotions in the struggle to maintain their position and protect themselves, and behave without empathy and compassion in relation to their colleagues and even the women under their care in the workplace. These changes may protect them more against STS, but they can also lead to neglecting the parturient during care provision. In such situations, a feeling of inner turmoil is occasionally created in the midwife that causes dissatisfaction and sense of inability to provide high-quality care to women and weakens the professional belonging and might even make them think about leaving the midwifery profession altogether [47]. In this regard Pezaro et al. called on the international community to make a universal innovation to protect and empower midwives in the workplace. She described the condition of a traumatized midwife as follows:

“‘Maternity Service Ship’ sails on, proudly flying the flag of being ‘with woman,’ look out for those who have been left behind, silently shouting ‘Midwife overboard’”[48].

### Risk management

Risk management was the third theme of the present study. This theme mainly indicates the reactions and strategies to cope with the incident that the participants used individually or in combination to reduce the probability of future risks. According to Endler and Parker’s (1994) theory, people’s style of coping with stress plays an important role in their cognitive adaptation and mental health. Studies show that people who focus on problem-solving skills are less likely to experience turmoil in stressful situations, have more control over them and are also better able to adapt to difficult situations than those who use emotion-focused coping and avoidant coping [49]. In this regard, although the adverse consequences of such events often diminish over time as midwives adapt to the risks, the actual manner of coping with these conditions can lead to reactions such as excessive fear and avoidance of repeated hazards or attempts to predict the traumatic situation and prevent the occurrence of danger, such as reviewing the root causes of accidents, learning and sharing experiences with peers, improving personal technical knowledge, evidence-based performance and ultimately self-empowerment. Hadjigeorgiou et al. also suggested in their qualitative study that the first step in advancing the midwifery profession is having access to continuing vocational training to teach midwives some key skills and further empower them in supporting mothers [32].

The positive aspects of task oriented-coping behaviors in this study included the positive experiences derived from accidents and trying to improve communication with the patient, which have been reported to lead to the appreciation of life, spiritual changes, and an increased ability in the traumatized individual [20], which often make midwives more determined to support the patients. They use their experience to modify and improve their performance and cooperate more with their other colleagues, and thus feel that they can become better midwives and build stronger relationships with their patients [19, 22, 40]. Sharing these experiences in a structured, safe, and threat-free environment is invaluable and serves as a vital and safe gateway for personal coping. Nonetheless, in some cases, the risk management briefing sessions held by peers, and especially the superiors, are more destructive than constructive [43]. In some studies, after exposure to incidents, some midwives have not been able to take care of their health for reasons such as systematic pressures, leading to their withdrawal and even leaving the midwifery profession [2, 5].

## Conclusions

The present study was an in-depth analysis of midwives’ experiences regarding their exposure to accidents and adverse events occurring in maternity wards, which has been conducted for the first time in Iran. The results showed that, in addition to the traumatic nature of the experienced incidents, contributing factors such as organizational governance structure, and weak supporting laws weaken the role of midwifery professionals in hospitals and further facilitate STS. In the meantime, the most important measures for reducing the physical, psychological, and social damage caused by STS that midwives can take to survive and improve their position are strategies for self-empowerment by learning about the existing professional rules, problem-oriented strategies such as empowering midwives for managing high-risk deliveries, educating and promoting the use of meditation to reduce the effects and symptoms of distressed midwives, and finally implementing the rules that support midwives more and can be trusted and referred to in case of traumatic events. To this end, it is necessary for policymakers in the area of maternal care to redefine, with the participation of stakeholders, the position and job description of midwives and enact laws to protect them and increase their professional authority in providing care services to patients.

### Limitations

Due to its qualitative nature, the results of this study cannot be generalized to all midwives working in the Iranian healthcare system, although generalizability is not the goal of a qualitative study as much as it is in quantitative studies. Hence, extensive quantitative research on the prevalence of STS among midwives working in maternity wards is necessary. Also, the interval of time from the incidents to the conducting of this study (recall bias) and the psychological-emotional differences among the interviewees may have affected their perception and understanding of secondary traumatic stress.

## Data Availability

The datasets generated during and analyzed during the current study are not publicly available due to [ Confidentiality of participants’ workplace names] but are available from the corresponding author on reasonable request.
